# Formal guidelines from an expert panel: intensive care unit medical staffing, organisation and working hours to improve quality of life at work in France

**DOI:** 10.1186/s13613-025-01432-4

**Published:** 2025-01-20

**Authors:** Nicolas Terzi, Guillaume Thiery, Nicolas Bèle, Naike Bigé, David Brossier, Alexandre Boyer, Edouard Couty, Laëtitia Flender, Cyril Manzon, Jean-Paul Mira, Sofia Ortuno, Vincent Peigne, Marie-Cécile Poncet, Sylvain Renolleau, Jean-Philippe Rigaud, Bérengère Vivet, Khaldoun Kuteifan, the French Intensive Care Society (FICS), the French National Council of Intensive Care Medicine (CNP MIR, Conseil National Professionel de Médecine Intensive Réanimation)

**Affiliations:** 1 CHU Rennes, Intensive Care Unit, Hôpital Pontchaillou, Université de Rennes, INSERM CIC 1414, Service de Médecine Intensive – Réanimation, 2, Rue Henri Le Guilloux, 35033 Rennes Cedex 9, France; 2 Service de Médecine Intensive Réanimation, CHU de Saint Étienne, Saint Priest en Jarez, France; 3 Université Jean Monnet, Research On Healthcare Performance RESHAPE, INSERM U1290, Université Claude Bernard Lyon 1, Saint Etienne, France; 4 Centre Hospitalier Intercommunal Fréjus Saint Raphaël, 83600 Fréjus, France; 5 Département Interdisciplinaire d’Organisation du Parcours Patient, Gustave Roussy, Service de Médecine Intensive Réanimation, 94805 Villejuif, France; 6 Pediatric Intensive Care Unit, CHU de Caen, 14000 Caen, France; 7 Medical School, Université de Caen Normandie, 14000 Caen, France; 8 ULR 2694 - METRICS : Évaluation des Technologies de Santé et des Pratiques Médicales, Université de Lille, CHU Lille, 59000 Lille, France; 9 Médecine Intensive Réanimation CHU Bordeaux, 33000 Bordeaux, France; 10 Chaire Santé de Sciences Po Paris, Paris, France; 11 Centre Hospitalier Universitaire de Reims, Reims, France; 12 Service de Réanimation, Médipole Lyon Villeurbanne. Service de Réanimation, 158 Rue Léon Blum, 69100 Villeurbanne, France; 13 Service de Médecine Intensive-Réanimation, Hôpital Cochin, Assistance Publique - Hôpitaux de Paris, 27 Rue du Faubourg Saint Jacques, 75014 Paris, France; 14 Université Paris Cité, Paris, France; 15 UMR 8104, Institut Cochin, INSERM U1016, CNRS, Université Paris Cité, 22 Rue Méchain, 75014 Paris, France; 16 Service de Médecine Intensive - Réanimation Cardiologique, AP-HP, Sorbonne Université, Pitié Salpêtrière, Paris, France; 17 Service de Réanimation, Centre Hospitalier Métropôle-Savoie, Place Biset, 73000 Chambéry, France; 18 Assistance Publique – Hôpitaux de Paris, Hôpital Avicenne, Hôpitaux Universitaires de Paris Seine-Saint-Denis, Paris, France; 19 Réanimation et USC Médico-Chirurgicales Pédiatriques-SMUR Pédiatrique, CHU Necker-Enfants Malades, AP-HP Paris, France; 20 Faculté de Médecine, Université Paris Cité, Paris, France; 21 Médecine Intensive Réanimation, Centre Hospitalier de Dieppe, Dieppe, France; 22 Service de Réanimation Polyvalente-USIP, GH de La Haute-Saône, Vesoul, France; 23 Service de Réanimation Médicale GHRMSA, Hôpital Emile Muller 20 Av. du Dr Laennec, 68100 Mulhouse, France

**Keywords:** Medical staff, Intensive care unit, Organisation, Quality of work life

## Abstract

**Background:**

Intensive care units (ICU) are characterized by high medical assistance costs and great complexity. Recommendations to determine the needs of medical staff are scarce, generating appreciable variability. The French Intensive Care Society (FICS) and the French National Council of Intensive Care Medicine (CNP MIR, Conseil National Professionel de Médecine Intensive Réanimation) have established a technical committee of experts, the purposes of which were to draft recommendations regarding staffing needs in ICUs and to propose optimal organisation of work hours, a key objective being improved workplace quality of life.

**Results:**

Literature analysis was conducted according to the GRADE methodology (Grade of Recommendation Assessment, Development and Evaluation). The synthesis work of the experts according to the GRADE method led to the development of 22 recommendations in 6 field. The experts issued a strong recommendation associated with a high level of evidence which is that work organization be given priority during periods of permanent care, with a maximum 16 h of consecutive work permitted. For 21 other recommendations, the level of evidence did not allow GRADE classification, and led to the formulation of expert opinions. All recommendations and expert opinions were validated (strong agreement).

**Conclusion:**

The work in the intensive care unit and in the intermediate intensive care unit is multifaceted, both clinical and non-clinical, and must include at least the following continuity and quality for patient safety. This document provides a detailed framework to propose an optimal medical staff.

## Introduction

Intensive care medicine is concerned with management of patients whose survival prognosis is compromised due to potentially reversible acute visceral failures, often caused by multiple factors. It is structured into two main categories: intensive care units (ICU) and intermediate intensive care units. For the sake of clarity, the term “intensive care” encompasses both ICU and intermediate intensive care unit (IICU) beds.

Application of the techniques necessary for patient care (mechanical ventilation, renal replacement therapy, cardiovascular monitoring and explorations, circulatory support) must be performed in an especially dedicated unit, designed, organized, and structured specifically in terms of architecture, equipment, and personnel.

The French health system provides universal access to care. The state is the main payer and most health professionals are salaried, at least in the public sector, which is the majority in intensive care. ICUs are governed by laws that set out their practices. The organisational model for French ICUs is that of closed units with a medical doctor (MD) on duty 24 h a day, 7 days a week, as required by law.

Intensive care medicine is among the healthcare activities requiring authorization from the general director of the regional health agency according to article R6122-25 of the national public health code [1]. In this context, a set of laws was issued on 26 April 2022 which defined the conditions for the establishment of intensive care activity, setting out minimum regulatory obligations in terms of structure and organization [2]. Even though they specify the non-medical human resources of adult and paediatric critical care units, the law does not provide for the necessary medical human resources, while the imperatives imposed on intensive care physicians in terms of care, research, and teaching are numerous.

There are major differences between countries in terms of organization and staffing ratios (Wunsch H current op Crit Care 4), which makes it difficult to generalize from one country to another. Nevertheless, these factors are of crucial importance for both the psychological well-being of health care professionals and the outcome of patients. Closed ICUs, for example, are associated with a better prognosis than open ICUs (JAMA 5 Carson SS). Similarly, organizational characteristics of ICUs have been associated with patient outcome after abdominal surgery (JAMA 3 Pronovost).

This series of formalized expert recommendations represents an attempt to address the medical needs of ICUs so that they can ensure not only safety and quality of care, but also quality of life at work for the intensive care physician. In addition, we have taken advantage of the new French regulatory constraints excluding medical staffing to carry out evidence-based reviews on medical staffing that could be useful to resuscitators in organizations similar to France (closed ICUs with dedicated staffing by intensivists).

## Method

These recommendations are the result of the work of an organizing committee and a group of experts convened by the French Intensive Care Society (FICS) and the National Professional Council (CNP) of Intensive Care Medicine. Initially, the organizing committee defined the questions to be addressed. It then designated experts in charge of each question. The questions were formulated in a PICO format (Patients Intervention Comparison Outcome) after an initial meeting of the expert group. Literature analysis was conducted according to the GRADE methodology (Grade of Recommendation Assessment, Development and Evaluation). A level of evidence was defined for each of the cited bibliographic references, depending on the type and methodological quality of the study. A high overall level of evidence enabled formulation of a “strong” recommendation (should be performed…GRADE 1 + , should not be performed…GRADE 1-). A “moderate”, “weak”, or “very weak” level of proof led to formulation of an “optional” recommendation (should probably be performed…GRADE 2 + , should probably not be performed…GRADE 2-). When the literature was non-existent or insufficient, the question could be subject to an expert opinion recommendation (the experts suggest…).

The proposed recommendations were presented and discussed one by one at a second expert meeting. Each recommendation was then evaluated by each expert and given individual ratings using a scale ranging from 1 (complete disagreement) to 9 (complete agreement). The collective rating was established according to the GRADE grid methodology. To validate a recommendation on a given criterion, at least 50% of the experts had to express agreement and fewer than 20% disagree. For an agreement to be strong, at least 70% of participants had to express agreement. In the absence of strong agreement, the recommendations were reformulated and, resubmitted for rating with the aim of reaching a consensus. The synthesis work of the experts according to the GRADE method led to the development of 22 recommendations. The experts issued a strong recommendation associated with a high level of evidence (GRADE 1 +). For 21 other recommendations, the level of evidence did not allow GRADE classification, and led to the formulation of expert opinions. All recommendations and expert opinions were validated (strong agreement).

Table 1: Recommendations according to the GRADE methodology.

**Table 1:** summary of recommendations

Field	Recommendation	Grade
*Field n°1* Activities of the intensive care physician	R1. Physicians engaged in full-time intensive care should be required to carry out two half-days per week of non-clinical work	Expert opinion, Strong agreement
*Field n° 2* Number of intensive care doctors required in the ICU with predominantly unscheduled activity	R1. Patients-to-physician ratio in during the day: 1 physician per 6 beds maximum in ICUs, and 1 physician per 8 beds maximum in intermediate ICUs	Expert opinion, Strong agreement
	R2. During night shifts and on weekends: one intensive care physician for a maximum of 14 intensive care beds in the ICU	Expert opinion, Strong agreement
	R3. One intensivist should be dedicated to daytime clinical activities outside the ICU	Expert opinion, Strong agreement
*Field n°3* Organisation of working time	R1. Work organisation should be optimized to limit the number of intensivists taking care of a patient during its ICU stay in order to promote continuity, quality and safety of care	Expert opinion, Strong agreement
	R2. The number of consecutive hours worked in the ICU should not exceed 16 h	Grade A Strong Agreement
	R3. The number of night shifts per month should be limited at 4 per month	Expert opinion, Strong agreement
	R4. Two shifts should be at least three days apart in order to reduce the risk of burnout or depressive syndrome	Expert opinion, Strong agreement
*Field n°4* Calculating working time	R1: The method used for precisely calculating physician worktime should take into account their activity in view of reducing burnout risk	Expert opinion, Strong agreement
	R2: On-call night shifts should be counted as three half-days or calculated in hours	Expert opinion, Strong agreement
	R3: It should be possible for an intensivist to telework for teaching, research or administrative tasks	Expert opinion, Strong agreement
	R4: The conditions for implementing teleworking for intensive care physicians must comply with the legislation	Expert opinion, Strong agreement
*Field n°5* Impact of the structure	R1. The same ICU physician-bed ratio for bedside working time in public and private hospitals with mainly unscheduled activity	Expert opinion, Strong agreement
	R1bis. Physicians in private hospitals must be free to organize their non-clinical working time	Expert opinion, Strong agreement
*Field n°6* Impact of age on career	R1: The experts are not able to recommend an age limit for participation in on-call duty	Expert opinion, Strong agreement
	R2: Consideration should be given to the possibility of adapting and/or diversifying, sometimes even temporarily, the ways in which work is carried out during a career in intensive care	Expert opinion, Strong agreement
	R3: The adaptation or diversification of a physician’s activity should not be compulsory but be personalized	Expert opinion, Strong agreement
	R4: Physician periodic health assessments should be carried out by the occupational medicine departments of the hospital	Expert opinion, Strong agreement
	R5: Physician should take part in the periodic monitoring organized by their hospitals’ occupational medicine departments	Expert opinion, Strong agreement
	R6: Awareness-raising/training in the screening of physical and cognitive disorders should be systematically and periodically provided to physicians involved in on-call duty	Expert opinion, Strong agreement
	R7: The experts suggest that priority should be given to considering ways of diversifying professional activity, such as limiting participation in on-call duty, particularly at night; setting up part-time or shared activity; moving towards transversal institutional or management activities, developing intensive care activities such as post-intensive care follow-up	Expert opinion, Strong agreement

## Results

### Field 1: activities of the intensive care physician

**Table:** 

R1: The experts suggest that physicians engaged in full-time intensive care in an intensive care unit, and who also host graduate and/or postgraduate students, should be required to carry out two half-days per week of non-clinical work. (Expert Opinion: Strong Agreement)	

### Rationale and reminders about the status of hospital practitioners

The majority of intensive care physicians in France are employed as hospital practitioners in public hospitals. Consequently, they are subject to a legal status that is analogous to that of all physicians employed in public hospitals. This framework provides the context for the description of activities.

#### Hospital practitioner (consultant) status

In accordance with the French Public Health Code, Hospital Practitioners (consultants) are responsible for carrying out defined functions in public health hospitals. They perform medical acts of diagnosis, treatment and emergency care, as well as non-clinical activities. A reform of consultant status came into effect on 6 February 2022. The objective was to facilitate the diversification of activities between public hospital activities, shared activities among healthcare or medico-social structures, and private practice, whether within their establishment or not, in order to open up career paths, thereby enhancing the attractiveness of hospital careers. The service obligations for full-time practitioners are 10 half-days per week, while part-time practitioners are required to work between 5 and 9 half-days per week.

Working hours for practitioner:

In accordance with Article R. 6152-27 of the Public Health Code, it is stated that: weekly working hours are set at ten half-days, with the understanding that the total number of hours worked per week shall not exceed forty-eight hours, calculated on average over a four-month period [4]. If the above activities are carried out during the night (from 18.30 to 8.30), they will be counted as two half-days. Saturday morning is considered a weekly working time. Saturday working hours are from 12 noon Saturday to 8:30 a.m. Sunday (equivalent to three half days) and Sunday working hours are from 8:30 a.m. Sunday to 8:30 a.m. Monday (equivalent to four half days).

In the event that medical activity is conducted on a continuous basis, the weekly service obligation of the practitioner is, by exception, calculated in hours on average over a four-month period and cannot exceed forty-eight hours a week. If practitioners opt to undertake additional work above and beyond their weekly service obligations, they may do so in one of two ways: either by taking time off in lieu or by receiving a continuity of care allowance and, if applicable, an additional work time allowance. A minimum daily rest period of eleven consecutive hours per twenty-four-hour period is mandatory. Practitioners may engage in continuous work for a maximum duration of twenty-four hours. In that case, they are entitled to an equivalent rest period.

#### Non-clinical valencies

In addition to their clinical roles, HPs may also fulfill non-clinical roles. These roles may involve contributing to teaching and research, holding institutional or managerial responsibilities, participating in collective projects, and structuring relations with primary care medicine. These activities are conducted within the institution.

#### General interest activity (AIG) external to be assigned

AIGs serve a general interest in healthcare, teaching, research, vigilance actions, networking, advisory or support missions. They must be carried out in a structure other than the practitioner’s main hospital.

#### Leave: practitioners are entitled to

- Annual paid leave, of which the duration is defined based on twenty-five working days, proportional to weekly service obligations;
- A paid leave of 20 days for working time reduction under the conditions set out in article R6152-801 [5];
- Training paid leave of fifteen working days per year, to update their knowledge.

#### Missions of a hospital practitioner in intensive care

- Care Activities: The medical work in intensive care and multipurpose intensive care units is divided into care activities, training (medical and paramedical), research, administrative tasks, and/or management. Care activity is the most significant part of the intensive care physician's mission in terms of purpose and time spent.

Care activities include:

- Multiple daily visits to patients
- Medical supervision of intra-hospital transports to imaging, interventional radiology, or the operating theatre
- Receiving and informing families
- Production of medical correspondence related to the patient's stay
- Medical consultation outside the department (hospitalization units, emergency departments)
- Participation in multidisciplinary consultation meetings
- Management of in-hospital life-threatening emergencies (such as blue code systems)
- Participation in patient follow-up after their stay in intensive care
- Management of technical cross-sectional activities (e.g., setting up vascular accesses)

b) In-hospital on-call periods

Operation of a complete line, 365 days a year, mobilises a significant number of full-time equivalent practitioners, taking into account training time and non-attendance, to cover the periods of in-hospital on-call periods care in healthcare establishments. Indeed, each on-call line mobilises a considerable volume of medical time, which must be remunerated, and mainly comes at the expense of the time available for daytime activities.

c) Training and Teaching:

Training activities are proposed to the medical staff of the unit throughout their professional careers. Some activities are specific to the hosting of students undergoing training. In light of the expectations of the medical training and research units (UFR) and given the increasing number of students to be trained, the training of students has become increasingly important.

d) Clinical Research:

Clinical research encompasses participation in research projects initiated by external sponsors, whether they be industrial or academic, as well as the development and implementation of research projects initiated by the unit itself. The representation of intensive care in international scientific publications is sizable, with an impact factor twice the global average (Scientific Production of University Hospitals 2006–2015). As evidenced by the National Council for Research Coordination (2018), commitment of its physicians to research is likewise considerable [3].

### Field no. 2: number of intensive care doctors required during the day for care activity in the intensive care platform with predominantly unscheduled activity

**Table:** 

Patient-to-physician ratio in intensive care units (including mainly unscheduled activity) Fig. 1)	
R1: Experts suggest 1 physician per 6 beds maximum	
Patient-to-physician ratio in intermediate intensive care units (mostly unscheduled activity)	
R1 Bis: Experts suggest 1 physician per 8 beds maximum Fig. 1)	

The patient-to-physician ratio (PPR) across intensive care units (ICUs) is not standardized and the association of PPRs with patient outcome is not well-established [6, 7]. The methodology for calculating the PPR varies depending on the study (declarative, observational, database). Generally, it does not correspond to the number of beds “managed” by an intensivist, but rather to the number of patients managed by an intensivist over a period of time (for example if an intensivist manages eight full beds from which two patients are discharged and to which two others are admitted on the same day, the PPR is 8 + 2 patients / 1 intensivist, i.e. 10, over that specific day).

Considering their internal validity, all of these studies are observational and explore the association between an endpoint (primarily patient mortality [8–15] or less frequently intensivist burnout [16, 17]) and exposure to a workload reflected by the PPR.

The difficulties arise firstly from the choice of this primary outcome, which is questionable. A multicentre observational study conducted in UK from 2010 to 2013 including almost 50,000 patients reported a median PPR of 8.5 [IQR 6.9–10.8; Range 1.0–23.5]. After multivariate adjustment, the PPR of each patient was significantly associated with hospital mortality (p = 0.003) [12]. Mortality appears to be modifiable only in the most extreme situations of PPR and does not accurately reflect quality of care, particularly concerning reduced ICU or hospital length of stay. Intensivist burnout is not the subject of appropriate studies.

In addition, exposure is not always clear. Should the PPR be calculated at the bedside or by taking into account the more global tasks of an intensive care doctor? Should it be calculated during the day or by mixing day and night? Should the doctors included in the ratio be restricted to senior intensivists, or extended to intensivists in training or organ specialists? Finally, confounding factors (severity of the case mix, nurse/patient ratio, other variables being potentially associated with the PPR) are not well accounted for in the models).

In terms of external validity, US-Canada studies [12–15, 18] are almost unusable because of their typical open-model multidisciplinary rounding ICUs, where the intensivist coordinates the specific tasks of other medical and non-medical specialists with advanced skills (pulmonologist, nephrologist, clinical pharmacist, etc.…). In these studies, the PPR appears high on a theoretical level (generally between 7.5 and 12) but the actual PPR is closer to 3–5 if one takes into account the different medical stakeholders. Regarding European studies (Switzerland, Netherlands [13]) the same problem of extrapolation arises. The best external validity comes from a French retrospective single-centre cohort study carried out on several Lyon ICUs in 2013 [10]. It included 5718 patients and assessed the association between PPR measured by 6-h shift and mortality adjusted in a Poisson model for patient turnover, burden, and percentage of medical vs. surgical patients. In this study, the PPR ranged from 3.2 to 9.5 and the daytime PPR was 3.6 on average. The only significant association in favour of excess patient mortality was observed when the PPR exceeded 14 compared to a PPR < 8.

### Physician-bed ratio on weekends and during on-call care:

**Table:** 

**Fig. 1:**
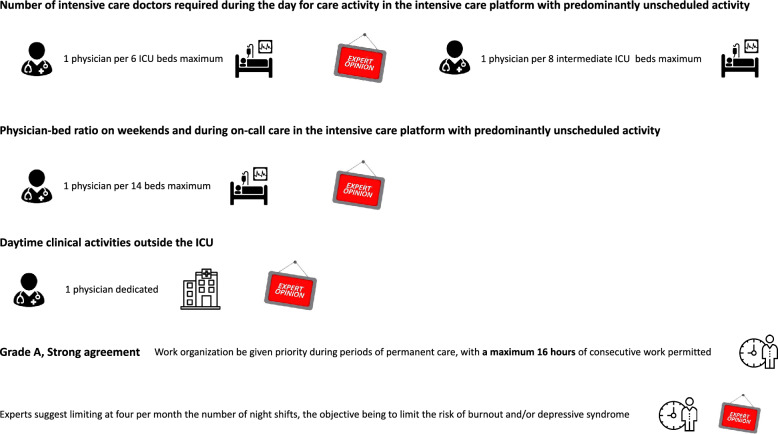
Number of intensive care doctors required during the day for care activity in the intensive care platform with predominantly unscheduled activity

R2: The experts suggest one intensive care physician for a maximum of 14 intensive care beds in the intensive care platform with predominantly unscheduled activity during night shifts and on weekends. (Expert opinion, Strong Agreement) (Fig. 1)	

### Rationale

Previous studies attempt to establish an association between morbidity and mortality rates in intensive care units (ICUs) and the size of medical staff [19, 20]. It is well-established that morbidity and mortality rates in intensive care units (ICUs) are influenced by the latter[19, 20]. For several decades, there has been a recurring pattern of excess mortality when admission to intensive care occurs on weekends compared with weekdays [21–23]. Since the 1970s, this phenomenon has been known as the “weekend effect” [20, 24]. A number of explanations have been put forth to account for these observations. Patients’ condition has been identified as the primary cause [20, 24]. It is possible that patients treated during the weekend have proportionately more severe illnesses, which may result in higher mortality rates [25]. In addition, the literature has focused on an organisational cause. A meta-analysis conducted by Galloway et al*.* in 2018 included 90,255 patients from 16 cohort studies across the globe. The analysis demonstrated that the “weekend effect” was observed exclusively in studies conducted in North America. The study did not find evidence of an excess mortality rate in studies conducted in Asia and Europe [23]. In a longitudinal study comprising 5718 stays in 2013 across eight adult ICUs at the French university hospital of Lyon, Neuraz et al*.* observed that above a patient-to-intensivist ratio of 14:1, the risk of mortality increased twofold (1.3–3.2). In this study, the reference ratio was 8:1 [26].

A series of studies conducted in Europe indicate that a minimum ratio above which mortality increases should be established [12, 13]. A review of the literature revealed no studies that specifically examined the impact of the patient-to-intensivist ratio on weekends. Consequently, the experts’ conclusions of are extrapolated. Nevertheless, the issue of continuity of care during the week and weekends has been the subject of specific studies. Ali et al. conducted a prospective, randomised study in alternative clusters to investigate the impact of a continuous organisational structure on medical activity (15 consecutive days including weekdays and weekends) on patient outcomes (mortality and length of stay), and on professional outcomes (burnout and stress), compared with an organisational structure that did not include working during the weekend. The study, which included 45 intensivists and 1900 patients, demonstrated that continuous organisation had a detrimental impact on professional outcomes without improving patient outcomes. Indeed, there was a discernible tendency for outcomes to worsen (adjusted mortality in intensive care: The odds ratio was 1.43 (95% CI 0.91–2.24), with a p-value of 0.12. [27]. The findings of this study reinforce the need to ensure the preservation of weekend periods for healthcare professionals continuously involved in the management of patients in the ICU.

**Table:** 

R3: The experts suggest that an intensivist should be dedicated to daytime clinical activities outside the ICU in order to decrease morbi-mortality of ICU patients admitted from the wards and to reduce burn-out risk among intensivists (Expert Opinion: Strong Agreement) (Fig. 1)	

### Rationale

In recent years, there has been a noticeable improvement in the prognosis of patients due to early detection and anticipation of organ failures, as well as greater involvement of intensivists outside ICUs. As a result of overall improvements in life expectancy and the greater complexity of medical conditions, clinical activities outside the ICU are increasing and adding significantly to ICU workloads [28–32].

The number and the nature of an intensivist’s outside tasks and the corresponding workload depend on local organization and on the participation of intensivists in interprofessional collaboration networks. A non-exhaustive list of intensivists’ clinical activities outside ICUs is provided in the following Table.

**Table:** 

ICU bed management	
Clinical evaluation of indications to ICU admission	
Rapid response team	
Early management of critically ill patients in general wards before ICU admission	
Invasive procedures (US-guided paracentesis, for example) at the bedside in general wards	
Invasive procedures for general ward patients in the ICU or a dedicated unit: chest drainage, bronchoscopy, central venous catheter placement	
Collegiality: medical advice for general ward patients, multidisciplinary team meetings, anticipated organ donation approach …	
Collegial ethics	

Excess workload resulting from addition of outside and inside activities potentially results in decreased quality, continuity and safety of care and consequently, in increased morbi-mortality. Complexity of interprofessional communication and relationships in outside clinical activities is time-consuming and potentially stressful [32, 33]. Moreover, increasing workload and task interruptions induced by outside activities may compromise quality and time dedicated to communication with ICU-team, ICU-patients and their relatives.

A non-exhaustive list of negative consequences of excess workload resulting from accumulation of outside and inside activities is proposed in the following Table.

**Table:** 

Task interruption	
Hurried and inappropriate decisions	
Delayed management of critically ill patients (inside or outside the ICU)	
Delayed ICU admission	
Impaired communication between intensivists and patients and their relatives (shorter time, altered quality)	
Impaired communication in ICU team: collegiality, clinical handover	

Excess workload, alteration of quality of working life, alteration of quality and safety of care and subsequent conflicts may increase the risk of intensivist burnout [16, 34–39].

As of yet, no study has compared different ICU organizational models with or without intensivists dedicated to outside clinical activities. Nevertheless, a French retrospective study showed that implementation of a rapid response team (RRT) led by an ICU physician was associated with reduced mortality [40]. The study does not specify whether or not the RRT leader was dedicated to RRT. Three meta-analyses have analyzed the interest of rapid response systems. Two of them confirmed their beneficial effect on survival [41, 42] and on incidence of intra-hospital cardiac arrest [42]. In the most recent meta-analysis, the effect of rapid response system on mortality, unplanned ICU admissions, hospital length of stay and, adverse events was weak or nil. However, this meta-analysis included four randomized trials in healthcare systems different from the French one [43].

### Field n°3: organisation of working time

**Table:** 

R1: The experts suggest that the number of consecutive working days should not exceed five out of seven, the objective being to improve quality of working life and to reduce burnout risk among intensivists. (Expert opinion, strong agreement). R2: The experts suggest that work organisation should be optimized to limit the number of intensivists taking care of a patient during its ICU stay, the objective being promote continuity, quality and safety of care. (Expert opinion, strong agreement).	

#### Rationale

In France, duration of work for a full-time intensivist is ten half-days a week with a legal limit of 48 h per week averaged over a four-month period [44].

Even though several studies have indicated that a high number of consecutive working days is associated with an increased risk of burnout among intensive care unit (ICU) physicians, no critical threshold has been identified. [16, 35, 45–47]. In any case, it is imperative that continuity of care be maintained in order to guarantee quality and safety of care. The optimal number of consecutive workdays that would be conducive to good quality of working life and a low risk of burnout, while ensuring continuity, quality and safety of care, is not yet known.

A randomised trial compared two staffing models in five American academic ICUs. The first was a continuous schedule, which involved daily coverage by a single intensivist over a period of half a month. The second was an interrupted schedule, which involved weekday coverage by a single intensivist, with weekend cross-coverage by colleagues. The interrupted schedule group exhibited lower burnout levels, reduced work–home life imbalance, and lower incidence of job distress. However, no significant differences were observed in ICU and hospital length of stay or in ICU and hospital mortality. A computer-based simulation study was conducted to model the impact of four different intensive care unit (ICU) staffing models on continuity of care, number of weeks of service, free weeks and free weekends. The “7 days on schedule” model, which included seven consecutive days of daily work, was compared to three shared daytime and nighttime service models, which included no more than three consecutive days of daily work and two on-call night shifts over a two-week period. The three shared service models were associated with increased continuity of care and a higher number of free weeks, free weekends and free days between clinical obligations [48].

Gershengorn et al*.* retrospectively analyzed the association between the number of consecutive days worked by intensivists and patient outcomes in 109 ICUs in Australia and New Zealand. ICU length of stay was shorter in ICUs where intensivists worked three consecutive days or less. However, these units represented only 15.6% of the ICUs included in the study. Compared to working seven consecutive days, working three consecutive days or less was associated with shorter ICU length of stay, fewer ICU readmissions and lower hospital mortality [49].

An American observational study conducted among 4826 resident physicians reported that working more than 48 h per week, which would correspond to a 5-day work week, was associated with an increased risk of medical errors, adverse events, attentional failures and crashes [50].

**Table:** 

R3: It is recommended that work organization be given priority during periods of permanent care, with a maximum 16 h of consecutive work permitted. (Grade A, Strong Agreement) (Fig. 1)

Continuity of care refers to the continuous availability of healthcare services to ensure patient care outside of regular opening hours. For a number of years, in view of minimizing the risks of medical errors due to fatigue and of ensuring patient safety healthcare professionals have been subject to strict limits on the number of consecutive working hours. However, the recommended number of consecutive working hours to ensure continuity of care has yet to be clearly defined, even though some general recommendations suggest limits, such as not exceeding 12 to 16 consecutive hours of work.

Many studies have shown that fatigue associated with demanding work can be linked to reduced well-being and increased occurrence of medical errors, which can compromise patient safety. Reducing the number of consecutive working hours aims to mitigate these detrimental effects.

Literature analysis is complicated by varying national regulations and organizations concerning intensive care provision across countries. The majority of North American studies have focused on the nighttime work of residents, drawing attention to the specificities of this category of physicians in training.

Since 2008, the National Academy of Medicine in the USA has evaluated the link between workload and patient safety [51], demonstrating that it is unsafe to work more than 16 h without sleep, and since July 1, 2011 limiting the working time of first-year practitioners on shifts to 16 consecutive hours or less.

An analysis of the effects of capping the duration of consecutive working hours showed a reduction in the risk of road accidents by 24% and of attentional errors by 18% [52]. In 2010, a systematic literature review [53] evaluated the effect of capping shifts at 16 h, revealing improved patient safety and physician quality of life in most analyzed studies.

In 2004, Landrigan et al*.* [54] conducted a prospective randomized study in ICUs comparing the occurrence of medical errors made by residents while working either 24-h shifts or fewer than 24 h. Residents in the > 24-h group (p < 0.001) made 35.9% more serious errors. Similarly, Lockley et al. [55] found a 50% reduction in attention errors when working shifts were set at < 16 consecutive hours compared to ≥ 24 h (P = 0.02).

Rahman et al*.* [56] showed in a pediatric ICU that working periods equal to or longer than 24 h every three or four days versus equal to or shorter than 16 consecutive hours were associated with significantly more attention errors and reported serious medical errors.

The literature also contains studies with contrasted results. For instance, Parshuram et al*.* [57] evaluated the effects of three different work schedules (12, 16, and 24 h) applied to intensive care residents, and found no difference regarding adverse events, residents' well-being, or continuity of care. Similarly, a multicenter randomized cross-over trial in pediatric ICU residents found that residents made significantly more serious medical errors during the intervention programs (< 16 h) with a relative risk of 1.53. However, after adjusting for patient-to-physician ratio, there was no difference between the two groups.

**Table:** 

R4: Experts suggest limiting at four per month the number of night shifts, the objective being to limit the risk of burnout and/or depressive syndrome (Expert Opinion, Strong Agreement) R5: Experts suggest that two shifts should be at least three days apart the objective being to reduce the risk of burnout or depressive syndrome (Expert Opinion, Strong Agreement)	

#### Rationale

Practitioners working in ICUs are particularly exposed to the risk of burnout, stress and depressive syndrome [36, 58–61]. Embriaco et al*.* reported rates of severe burnout and depressive syndrome of 46.5% and 23.8% respectively [16, 62]. In these two surveys, carried out on the same cohort of intensive care physicians, the authors studied the factors favouring the onset of severe burnout and/or depressive syndrome and their link with the number and rhythm of night shifts [16, 62]. Practitioners identified as having a low level of burnout reported 4.6 (± 2) night shifts per month compared with 4.7 and 5.0 (± 2) for those with intermediate and high burnout scores respectively. Similarly, the frequency of on-call duty over the seven days preceding the survey and the length of time since the last previous week without work were independently correlated with burnout. The practitioners least exposed to the risk of burnout were those performing the closest to 4/month number of shifts with seven days between shifts [16]. The results with regard to the factors favouring the onset of depressive symptoms were comparable.

Without necessarily being responsible for burnout or depressive syndrome, consecutive hours of night work lead to drowsiness, stress, malaise, sleep disorders and impaired cognitive function (10–19). In a prospective study of 51 intensivists, Maltese et al*.* observed that regardless of a practitioner’s age or experience, all cognitive functions were impaired after night shift. They also found that the impairment persisted for an indefinite length of time, even after a few hours of sleep [63]. Furthermore, a prospective observational study showed that intensive care doctors report peaks of drowsiness and fatigue during their activities, both day and night [64]. These different symptoms can lead to a loss of empathy and a greater risk of medical errors. However, the data in the literature are insufficient to establish a precise recommendation on the number and frequency of on-call activities. Once again, disparities in hospital operations, local organization, resources and healthcare systems make it difficult to transpose results published by teams working in other countries to French practice. Furthermore, the literature is essentially made up of surveys aimed at evaluating the impact of night-time practice on the occurrence of individual events such as burn-out [16], a depressive syndrome [62], or fatigue with impaired vigilance [63–65]. Although evaluated using validated scales, these assessment criteria remain subjective and heterogeneous. We did not find any data enabling us to assess the consequences of an on-call model based on objective and criteria for judging morbidity and mortality or care-related complications. In the absence of robust data from the literature, it would seem safe to consider the factors frequently leading to the onset of burnout, depressive syndrome or impaired cognitive function in intensive care doctors. Among these factors, number and pace of night shifts could have a deleterious impact the quality and safety of care for intensive care patients.

The number of working hours, consecutive days at work and on-call on site duties have been identified as risk factors for burnout among intensivists. Quantifying intensivists’ working time of is therefore mandatory in view of limiting burnout.

### Field n°4: calculating working time

**Table:** 

R1: The experts suggest that the method used for precisely calculating intensivists worktime takes into account their activity in view of reducing burnout risk. (Expert opinion, strong agreement) R2: The experts suggest that on-call night shifts should be counted as three half-days or calculated in hours. (Expert opinion, strong agreement)	

#### Rationale

Calculation methods (“continuous medical time” or “half-days”) give very different results for the on-call periods. The “continuous medical time” method counts 24 h of work for a day followed by on-call duty, which corresponds to at least 50% of service obligations (since they cannot exceed 48 h per week). The same shift (one day followed by a night on-call) represents only 40% (4 half-days) of the service obligations with the “half-days” method.

A French physician who exceeds his/her weekly service obligations receives an extra remuneration, called “additional time”. Since on-call work is better taken into account, it is likely that the threshold for triggering additional time will be reached after fewer hours worked with the “continuous medical time” method than with the “half-days” method.

No published study has compared the impact of these two different methods of calculating working time on the risk of burnout and intensivist quality of life. While two US studies have evaluated different medical time organizations, they did not include on-call duties by intensivists.

Whichever method of calculation is chosen, a better accounting of working time will probably reveal the need to increase the number of staff needed to ensure service obligations, all the while respecting the maximum weekly duration of 48 h. According to a survey among 85% of French ICUs (299 ICUs) performed in 2021, the “continuous medical time” method was used in 55% ICUs and the switch from the “half-days” method to the “continuous medical time” method required an increase of staffing in 20% of them [66].

In turn, increasing the number of intensivists includes a risk of weakening team cohesion. Similarly, continuity of care may be more difficult to maintain with larger numbers of physicians. However, different organizational arrangements can be put in place to reduce these risks.

**Table:** 

R3: The experts suggest that it should be possible for an intensivist to telework for teaching, research or administrative tasks. (Expert opinion, Strong agreement) R4: The experts recall that the conditions for implementing teleworking for intensive care physicians must comply with the legislation. (Expert Opinion, Strong Agreement)	

#### Rationale

##### Teleworking, definition

Teleworking has been defined in the French Labour Code since July 2023. Article L.1222–9 defines it as any form of work organization in which work that could also have been carried out on the employer's location is carried out by an employee away from this location on a voluntary basis, using information and communication technologies [67].

##### In practice, in France

The French National Institute for Research and Safety (INRS) has published a summary of the use of telework in France. It points out that 2017 was a turning point, particularly with the democratization of ‘occasional’ teleworking. In France, teleworking is either regular, imposed by the employment contract at regular intervals (e.g. one day a week), occasional, on the occasion of a particular circumstance (‘personal convenience’, transport strike, childcare problem, etc.) or exceptional, imposed by the employer on the occasion of an exceptional event (e.g. natural disaster, bad weather, pandemic, etc.). In the executive population, 11% telework regularly and 15% occasionally [68].

##### In medicine

There are no data and framework for teleworking in the medical world in France. Resource management of medical skills is different in France and in North American countries. In Canada and United States, night duty is provided by a resident, who is in contact with a senior intensivist by telephone. This means that the qualified intensive care doctor is only on call at home [69, 70].

The North American system is also developing ‘intensive care teleconsultation’ systems, managed by a trained nurse, enabling early triage and targeted transfer of patients to a resuscitation sector, and on-site administration of first-line treatment [71, 72]. These arrangements appear to have a beneficial effect on patient flows, on quality of care and on nurses' job satisfaction. There are no data on the impact of this organization on doctors. We do not believe that the ‘emergency telephone service’ falls within the scope of teleworking, as it represents a very occasional aspect of support for a practitioner, which seems difficult to formalize.

In France, the doctor's tasks go beyond the clinical sphere. They extend to the fields of teaching, research and administration. These activities do not necessarily require the doctor to be present at the hospital, especially as there may be organizational constraints, such as the fact that each doctor now has an individual office (increased number of doctors without infrastructure improvements). It would therefore be conceivable for a doctor to carry out these tasks outside the hospital site. In the absence of data in the literature, it is not possible to impose this organization.

##### Elements to be respected in the practice of telework, lessons from the COVID-19 pandemic

While the COVID-19 pandemic imposed telework in many areas, it also highlighted the difficulties of this form of organization. It seems important to maintain a regular presence in intensive care units, thereby maintaining the social ties necessary for teamwork. The French Cour des Comptes has published a report on teleworking in the post-crisis period, noting that it is used by only 52% of hospital administrative staff [73]. The report also details number of processes for which teleworking is ineligible, including the handling of confidential data. A study by the French Ecole des Hautes Etudes en Santé Publique on the introduction of teleworking in hospitals ruled out the possibility of teleworking for medical staff, considering only the provision of care [74].

### Field n°5: the impact of the care structure

**Table:** 

R1. Experts suggest the same ICU physician-bed ratio for bedside working time in public and private hospitals with mainly unscheduled activity. (Expert Opinion: Strong Agreement) Physicians in private hospitals must be free to organize their non-clinical working time. (Expert Opinion: Strong Agreement)	

In France, the status of intensivists differs between private and public institutions. In contrast to the remuneration structure of public healthcare institutions, where doctors are not remunerated on a fee-for-service basis, private intensivists are self-employed and receive payment on a fee-for-service basis. Their remuneration is calculated on a daily basis and is dependent on two factors: firstly, the severity of the patient's condition and secondly, per eligible technical procedure. Nevertheless, as intensive care is regarded as an emergency (including that which follows planned surgery), the procedures are priced in accordance with the standard agreement with the French Primary Health Insurance Fund. Each procedure is attributed to a specific practitioner. It is typical for these remunerations to be aggregated into a unified fund per unit, which is then distributed among practitioners in proportion to their respective time allotments. This system results in a prioritization of working time at the patient's bedside. Private intensivists are not entitled to statutory time dedicated to work outside the clinic, nor are they provided with funding for their work time outside the ICU. Consequently, non-clinical activities (e.g. continuing medical education) are financed by the common pot. It is a contractual obligation for all physicians associated with a single ICU to engage in ongoing training. Furthermore, they are approved by their national organization (the National Union of Liberal Professions, UNAPL) and subject to scrutiny by insurance companies. Other activities of general interest, such as research, are conducted outside of clinical working hours and are not specifically funded. In light of the aforementioned considerations, it is imperative to devote particular attention to the specificities of ICUs in private institutions with regard to the patient-physician ratio. Indeed, incorporating non-clinical activities into overall working time, as is the case in public institutions, will, in mathematical terms, result in a reduction in the time dedicated to clinical activities. This will result in the recruitment of additional physicians to the ICU team, which will have the mechanical effect of diluting revenue per doctor and modifying the current economic model, with the potential risk of loss of attractiveness. It is therefore recommended that the objective be to achieve the same ratio of patients to physicians at bedside, in both private and public ICUs. Nevertheless, the overall patient-physician ratio could be calculated differently between the two institutions.

### Field n°6: impact of age on career

**Table:** 

R1: The experts are not able to recommend an age limit for participation in on-call duty. (Expert opinion, Strong agreement) R2: In view of the demands of intensive care work, particularly night work, and the effect of age on quality of care and doctors’ personal health, the experts suggest that consideration be given to the possibility of adapting and/or diversifying, sometimes even temporarily, the ways in which work is carried out during a career in intensive care. (Expert opinion, Strong agreement) R3: The experts suggest that the adaptation or diversification of a doctor's intensive care activity during his or her career should not be compulsory but be organized according to the doctor's state of health and his or her career plan, and that it be possible to re-evaluate this over time (Expert opinion, Strong agreement) R4: The experts recommend relying on the periodic health assessments carried out on doctors by the occupational medicine departments of their hospitals, on data relating to the periodic certification of healthcare professionals, on the periodic assessment of the quality of life at work and on practitioners' career plans. (Expert opinion, Strong agreement) R5: The experts recommend that doctors take part in the periodic monitoring organized by their establishments' occupational medicine departments. (Expert opinion, Strong agreement) R6: The experts recommend that awareness-raising/training in the screening of physical and cognitive disorders should be systematically and periodically provided to resuscitation doctors involved in on-call duty. (Expert opinion, Strong agreement) R7: The experts suggest that priority should be given to considering ways of diversifying professional activity, for example: (1) Limiting participation in on-call duty, particularly at night, or changing the conditions under which this is carried out; (2) Setting up part-time or shared activity; (3) Moving towards transversal institutional or management activities; (4) Developing intensive care activities such as post-intensive care follow-up (e.g. post-intensive care consultation, rehabilitation, etc.); (Expert opinion, Strong agreement)	

Given the low level of evidence in the literature, a recommendation based on the GRADE method cannot be made. Legally in France, the age limit for practicing medicine has been set at 72 since 2016 [75], partly in response to workforce issues within the profession. In 2022, this provision was extended until 31 December 2035 [76]. According to a recent survey the median age of intensivists is 43 (IQR 35–54), 36% of doctors are over 50 and almost 12% are aged 60 or over and therefore approaching retirement [66]. According to the Council of the American Medical Association, the number of practicing doctors aged over 65 increased by 374% between 1975 and 2015 and, in 2015, 23% of practicing doctors were aged 65 or over [77].

Regardless of the specialty in which they practice, ageing practitioners give rise to several issues. On the one hand, those of performance and quality of care, and on the other, those of quality of life at work, quality of personal life and practitioner’ state of health. The practice of intensive care medicine is particularly demanding, involving situations of tension and stress, repetition of difficult situations, and long shifts on call and during night shifts. As a result, we need to be able to guarantee the quality and safety of care for patients, while constantly considering the physical and cognitive state of health of practitioners throughout their career. However, while cognitive abilities change with age, there is considerable individual variability that needs to be considered when managing the ageing of professionals [77]. However, there are not enough studies examining the assessment of skills during the ageing process and the impact of burnout on older doctors [77].

The question of how old doctors are and when they should stop practicing has been controversial for many years. A growing body of evidence on the relationship between age and physician performance has led organizations such as the American College of Surgeons to address the issue [78]. There are reports of doctors continuing to practice at an advanced age despite impaired ability, but the profession has not demonstrated its ability to control this. Furthermore, setting a mandatory retirement age could be discriminatory and deprive many competent doctors of practicing their profession, and could lead to a shortage of doctors [77].

An Australian survey published in 2021 assessed the mental health of doctors in all age categories (49,596 questionnaires sent out—26.6% response rate). It was found that psychological stress, burnout and suicidal ideation were less common among older doctors (aged 61 and over). On the other hand, the risk of psychological distress was higher among older doctors suffering from a pre-existing mental health problem. Among the activities that caused the most stress for older doctors were long working hours and a workload that was considered too heavy. However, the older doctors had fewer financial stressors but more personal stressors (health problems, caring role, death in the family) than their younger colleagues. It was also suggested that doctors who practiced at an advanced age had probably developed greater resilience and, in fact, greater professional maturity [78]. This work highlights the need to consider the health of doctors as a function of age and career stage. According to a survey, the quality of life at work experienced by intensive care practitioners is very much affected by the number of on-call duties, and indeed by night work, regardless of age [79]. Continuity of care and, in particular, a high number of on-call duties are associated in the literature with an increased risk of burnout and medical errors, which can adversely affect patient safety and practitioners' quality of life. Among older practitioners, the recurrent fatigue associated with night work could have a currently unmeasured impact on practitioners' health that is [63]. A study carried out between 2018 and 2019 in the form of questionnaires among Chinese resuscitators highlights the fact that, given their conditions of practice, almost 69% of them expressed an intention to give up practising intensive care [80].

French law already provides a framework for the adaptation and reduction of care activities for older practitioners. It is possible to obtain a dispensation from the continuity of care at night for a practitioner aged over 60, on reasoned request and subject to service requirements. This derogation is granted by the director of the establishment, on the advice of the head of the unit and the committee responsible for organizing continuity of care [81]. Similarly, it is possible to obtain a reduction in weekly working hours, subject to service requirements [82]. Reducing working hours through part-time work is also a possibility governed by law [83]. It could also be suggested that participation in on-call duty be adapted in the form of a reduction in the number of on-call duties, half-shifts or daytime on-call duty at weekends and public holidays, etc.

So, as they develop their careers, it would seem advisable to offer practitioners a recurring health and activity check-up to suggest that they adapt and/or diversify their practices, either temporarily or permanently, according to their needs. Daily activity, on-call care and night work all contribute to Quality of Life in the workplace, a parameter that is now essential for all professionals to consider. However, in the case of a project to diversify professional activity in intensive care, care must be taken not to generate a feeling of discrimination or loss of salary for practitioners.

Other adaptations and/or diversifications of professional activity may be proposed, such as investment in cross-disciplinary institutional or management activities, or the development of less ‘stressful’ intensive care activities such as post-intensive care consultations [84] or participation in post-intensive care rehabilitation activities [85], even if, in the latter case, the issue of continuity of care may also arise.

The experts are therefore not able to recommend an age limit for participation in on-call duty. However, beyond a certain age, practitioners should be systematically offered the opportunity to be made aware of the need to screen for physical and cognitive disorders, to ensure their physical and psychological integrity, in their own interest and to guarantee that quality and safety of care is maintained. On the one hand, it seems essential to take account of the practitioner's personal health, as mentioned in the context of the Periodic Certification of Healthcare Professionals [86], and on the other hand, that the practitioner be involved in preventive actions concerning him or her.

## Conclusion

These guidelines contain a number of recommendations to both improve the quality of care and reduce the risk of burnout among ICU physicians. For daytime clinical activity, a ratio of one physician for six beds of less should in ICUs and one physician for eight beds of less in intermediate ICUs should be applied. At night times and weekends, a ratio of one physician for fourteen beds or less should apply. At least one physician should be dedicated for daytime clinical activities outside the ICU. Every intensivist working full time in an ICU should be required to work two half days per week on non-clinical work. The number of consecutive hours worked in the ICU should not exceed sixteen hours. The number of consecutive working days in the ICU should not exceed five days out of seven. The number of night shifts should be limited to four per month, with at least three days between two night shifts. Further studies in European ICUs are needed to improve the organization of work in ICUs.

## Data Availability

Not applicable.
